# Potential Sources of High Frequency and Biphonic Vocalization in the Dhole (*Cuon alpinus*)

**DOI:** 10.1371/journal.pone.0146330

**Published:** 2016-01-05

**Authors:** Roland Frey, Ilya A. Volodin, Guido Fritsch, Elena V. Volodina

**Affiliations:** 1 Leibniz Institute for Zoo and Wildlife Research (IZW), Berlin, Germany; 2 Department of Vertebrate Zoology, Faculty of Biology, Lomonosov Moscow State University, Moscow, Russia; 3 Scientific Research Department, Moscow Zoo, Moscow, Russia; Universite Paris XI—CNRS, FRANCE

## Abstract

Biphonation, i.e. two independent fundamental frequencies in a call spectrum, is a prominent feature of vocal activity in dog-like canids. Dog-like canids can produce a low (f0) and a high (g0) fundamental frequency simultaneously. In contrast, fox-like canids are only capable of producing the low fundamental frequency (f0). Using a comparative anatomical approach for revealing macroscopic structures potentially responsible for canid biphonation, we investigated the vocal anatomy for 4 (1 male, 3 female) captive dholes (*Cuon alpinus*) and for 2 (1 male, 1 female) wild red fox (*Vulpes vulpes*). In addition, we analyzed the acoustic structure of vocalizations in the same dholes that served postmortem as specimens for the anatomical investigation. All study dholes produced both high-frequency and biphonic calls. The anatomical reconstructions revealed that the vocal morphologies of the dhole are very similar to those of the red fox. These results suggest that the high-frequency and biphonic calls in dog-like canids can be produced without specific anatomical adaptations of the sound-producing structures. We discuss possible production modes for the high-frequency and biphonic calls involving laryngeal and nasal structures.

## Introduction

Biphonic vocalizations with two independent (high and low) fundamental frequencies in call spectra are common among dog-like canids: African wild dogs *Lycaon pictus* [1,2], Asiatic wild dogs or dholes *Cuon alpinus* [3,4], timber wolves *Canis lupus* [5–7], domestic dogs *C*. *lupus* f. familiaris [8,9], dingos *C*. *lupus dingo* [10] and red wolves *C*. *rufus* [11]. In contrast, biphonic calls are lacking in fox-like canids: red fox *Vulpes vulpes* [12–14], swift fox *V*. *velox* [15,16] and Arctic fox *V*. *lagopus* [17–19]. In red fox, a detailed analysis of 12,964 whines recorded from 75 individuals did not reveal one single biphonation [14].

**Fig 1:**
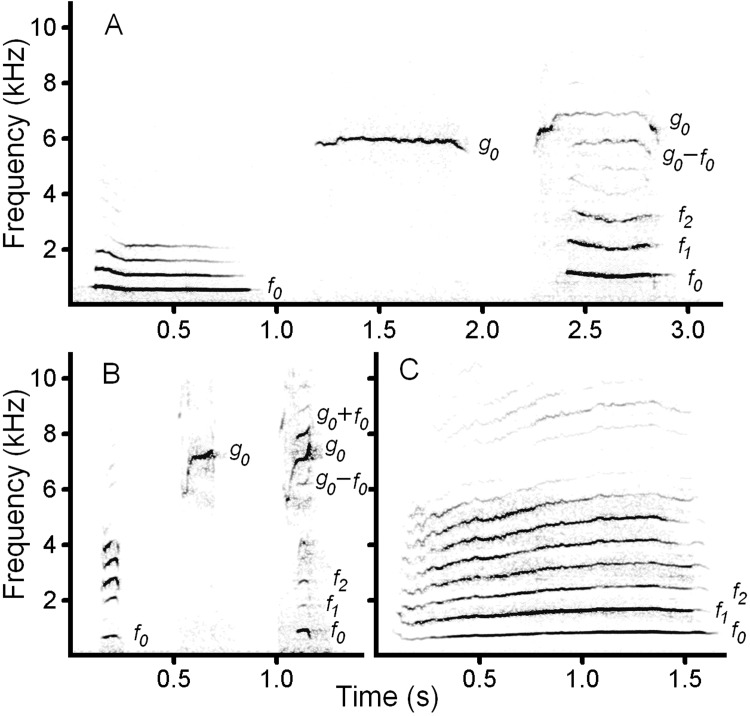
Spectrogram illustrating high-frequency and low-frequency vocalization modes in domestic dog (A), dhole (B) and red fox (C). (A) Dog (7-kg dachshund): left–low-frequency whine; middle–high-frequency squeak; right–biphonic whine-squeak (S1 Audio). (B) Dhole: left–low-frequency yap; middle–high-frequency squeak; right–biphonic yap–squeak (S2 Audio). (C) Red fox–low-frequency whine (S3 Audio). Designations: f0 –low fundamental frequency; f1 and f2 –harmonics of f0; g0 –high fundamental frequency; g0–f0 and g0+f0 –the linear combinations of f0 and g0. The spectrogram was created at 22050 Hz sampling frequency, Hamming window, FFT 1024, frame 50%, overlap 93.75%.

In domestic dogs, the range of the low fundamental frequency (f0) is 0.4–1.4 kHz and the range of the high fundamental frequency (g0) is 3.1–11 kHz [9] (Fig 1A). In the dhole, the range of f0 is 0.5–1.4 kHz and the range of g0 is 5.5–10.7 kHz. In red fox, the range of f0 is 0.32–1.21 kHz, while g0 is missing [13,14] (Fig 1C). The canid high-frequency calls are termed squeaks, whereas the low-frequency calls are historically termed whines in dogs and in red fox [9,13] but yaps in the dhole [4]. Dhole yaps are much shorter and sound differently compared to dog and fox whines. Biphonation takes place when the high-frequency squeak and the low-frequency whine or yap occur simultaneously. The simultaneously produced g0 and f0 interact with appearing additional frequency bands, representing linear combinations of the original frequencies. The additional frequency bands can be calculated by the formula n*f0 + m*g0, where n and m are integer multiples of f0 and g0 [1,3,4] (Fig 1B). Acoustic structures and frequency ranges are very similar between pure low-frequency calls (dhole yaps and dog whines) and the low-frequency components of the biphonic calls [3,4,8,9,20] (Fig 1A and 1B).

Experimental studies with anesthetized domestic dogs suggest that the f0 is produced by normal vocal fold oscillations [21,22]. Studies with dog-wolf hybrids and domestic dogs combined data on laryngeal anatomy and call acoustic structures [21,23–27]. A comparison of relative sizes of different parts of the vocal tract (vt) was done for red fox and domestic dogs [28]. Nevertheless, vocal anatomy of non-domestic canids is not yet investigated in detail [29–32] and production mechanisms for canid g0 remain unclear.

There are several explaining hypotheses for mammalian biphonation. The hypothesis of asynchronous vibration of the left and right vocal folds [21,33] is hardly relevant for the dhole. In biphonic calls of humans and nonhuman primates, this mechanism results in closely spaced f0 and g0 [34,35], whereas in biphonic calls of dholes and domestic dogs, the f0 and g0 are spaced widely [3,4,8,9].

The hypothesis of involvement of vocal fold extensions (vocal membranes) for the g0 production [23,36,37] applies to the Sykes’s monkey *Cercopithecus albogularis* [36] and to a single individual dog-wolf hybrid [23]. In canids, the presence or absence of vocal membranes has not yet been anatomically investigated. At the same time, former bioacoustical studies revealed biphonation in the total of 14 subject dholes [3] and in 8 of the 9 subject domestic dogs [9]. Therefore, if vocal membranes indeed represent the source for producing g0 in the dhole, they should be found in all individuals of this species without exclusion.

The hypothesis of g0 production by creation of vortices at the glottis or vt narrowings [1,22,34] has been proposed by [22]. These authors experimentally obtained from deeply anesthetized domestic dogs the oral whines and the biphonic whine-squeaks with g0 ranging of 3.2–3.7 kHz. A cineradiographic study of a non-anesthetized individual domestic dog revealed that during whining, it kept its mouth closed and lowered the soft palate to secure position of the laryngeal entrance in the nasopharynx, thereby passing the entire exhalatory air flow through the nose [38]. So, the narrowings potentially responsible for g0 production may occur not only in the oral but also in the nasal vt.

Biphonation might also result from source-filter interaction when the vocal folds start oscillating at one of the formant frequencies [34,39–41]. Source-filter interactions have been found in human singers [42], white-handed gibbons *Hylobates lar* [43] and in a single roar of an Iberian red deer *Cervus elaphus* stag [44]. Despite the rarity of this production mode, it cannot be excluded for the dhole.

**Fig 2:**
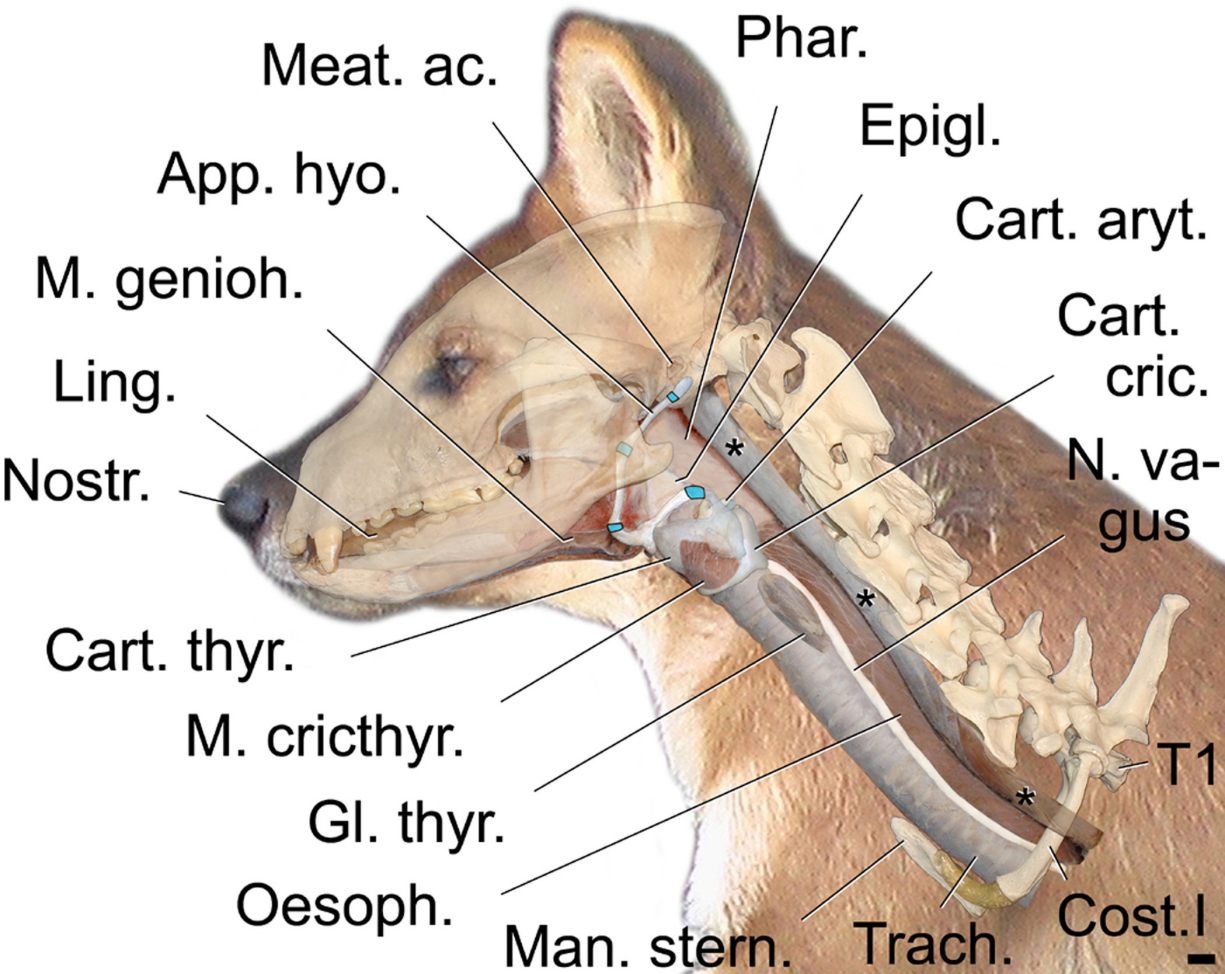
Gross morphology of the vocal apparatus, tongue and hyoid of an adult male dhole in near-natural position. Overlay reconstruction based on CT scans and macroscopic anatomical dissection. Skull, skeletal parts of the neck and thoracic inlet and contours of a typical head-and-neck posture of a live animal are provided in addition to facilitate an integrative view of the dhole vocal organs. Constrictor muscles of the pharynx removed. *** = lateral edge of neck musculature. Scale bar = 10 mm.

This study investigates the vocal anatomy of the dhole including the head-and-neck region (Fig 2), focusing on structures potentially capable of producing the high fundamental frequency (g0). In addition, we analyze vocalizations, collected during the life time of the same dhole individuals that served postmortem as anatomical specimens. For comparison, we briefly investigate the vocal anatomy of red fox as species which is not known to produce the high-frequency and biphonic calls.

## Material and Methods

### Ethics statement

No specific permissions were required for access to the locations and to the species of this study. The dholes used for the anatomical investigation had died from natural reasons in zoos; no one dhole was sacrificed for this study. The red foxes used for the anatomical investigation were shot in the course of legal hunting in Germany. Red fox is not an endangered or protected species in this country. The authors were not involved in this hunting. Vocalizations were recorded from outside the animal enclosures during zoo working hours. Hence, the call collectors were indistinguishable from common zoo visitors for the study animals. Disturbance of animals was kept to a minimum. The animals were not manipulated for the purpose of this study. As the two authors, who collected dhole vocalizations (IAV and EVV) are zoo staff members, no special permission was required for them to work with animals in the zoo. The research protocol # 2011–36 has been approved by the Committee of Bio-ethics of Lomonosov Moscow State University. We adhered to the ‘Guidelines for the treatment of animals in behavioural research and teaching’ (Anim. Behav., 2006, 71, 245–253) and to the laws on animal welfare for scientific research of the Russian Federation and Germany, where the study was conducted.

### Subjects

Dhole anatomical specimens were 1) a 12-year-old male whole-body fresh specimen (postmortem weight 21 kg) from Zoo Münster (Germany), born 11.11.1997, died 15.01.2008, deep-frozen; 2) a 15-year-old female whole-body fresh specimen (postmortem weight 11.5 kg) from Tierpark Berlin (Germany), born 17.04.1993, died 31.03.2008, deep-frozen; 3) a 8-year-old female head-and-neck specimen (postmortem weight unknown) from Volokolamsk Zoo Breeding Station (Russia), born 31.03.1995, died 06.08.2003, formalin-preserved; 4) an excised larynx of a 2 year-old female specimen (postmortem weight unknown) from Moscow Zoo (Russia), born 19.03.1999, died from accident 20.03.2001, formalin-preserved. In addition, we anatomically investigated a formalin-preserved whole-body specimen of a female dhole specimen, still-born 27.02.2002 in Moscow Zoo. Red fox specimens (one male, one female) were wild adult animals legally shot without damage of their head-and-neck regions on 06.12.2009 about 150 km to the south of Berlin city (Germany), deep-frozen.

### Anatomy and computer tomography (CT)

**Fig 3:**
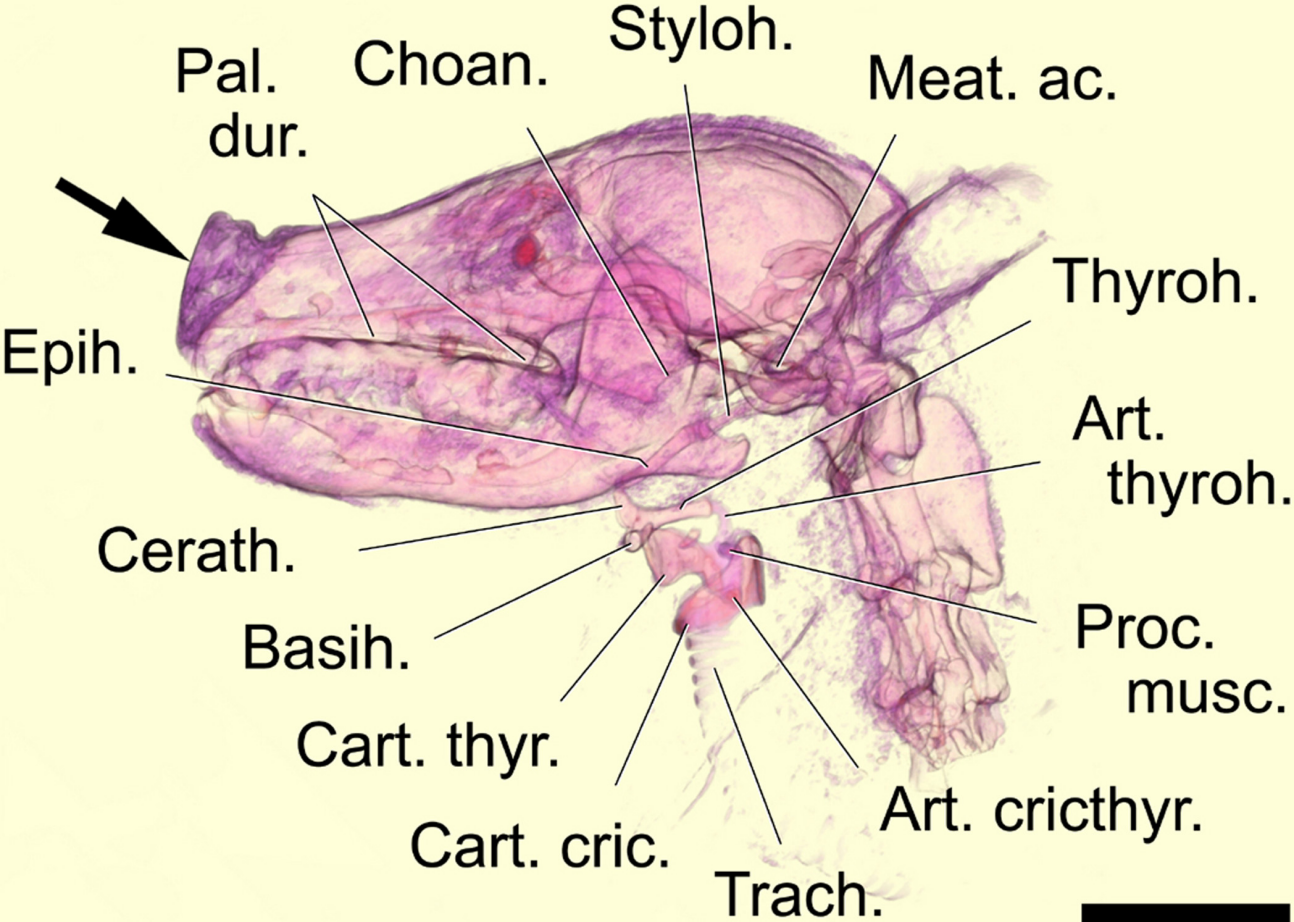
Computed 3-D reconstruction based on a CT scan of a female dhole head and neck specimen. In-situ resting positions of hyoid apparatus and larynx. Mediosagittal virtual section, right half, medial view. The arrow points to the flexible nostril region. Scale bar = 50 mm.

We conducted macroscopic dissections using binocular head loupes (Carl Zeiss Jena GmbH, Jena, Germany) with the specimens submersed in water. Measurements of vt length were taken with a string. Photos of successive steps of dissections were done with a Nikon D70S digital camera (Nikon Corp., Tokyo, Japan) and a 60 mm, 1,28 D, AF MikroNikkor-lens. Illumination for taking the photos was provided by 4 small digital flashlights (Metz 28 CS-2) plus 2 photo lamps (Paulmann halogen bulbs, 122 mm diameter, flood 30°, 230V, E27, 100 W). Photo images were processed with Photoshop (Adobe Systems, San Jose, California, USA). CT scanning was done with a 64-slice spiral Computer Tomograph Aquilion CX (Toshiba Medical Systems Corp., Shimoishigami, Japan) at the IZW. Postmortem *in situ* positions of the vocal organs were registered in black and white virtual serial sections (MPRs with settings 120.0 kB, 120.0 mA, slice thickness 0.6 mm) and in 3D-reconstructions with software Vitrea 2 (Toshiba Medical Systems Corp., Shimoishigami, Japan) (Fig 3). The CT data provided starting points for the further accurate anatomical dissections.

### Call collection and analysis

Spontaneously produced calls of 4 (1 male, 3 female) dholes were recorded in 2001–2003 in zoos of Russia and Germany. The same specimens were used postmortem for investigation of the vocal anatomy. For acoustic recordings (frequency range 40–15,000 Hz, distance to animals 2–8 m, after-recording digitizing at 48 kHz), we used a SONY WM-D6C cassette tape recorder (Sony Corp., Tokyo, Japan) and a Sennheiser K6-ME64 microphone (Sennheiser electronic, Wedemark, Germany). For simultaneous video recordings of calling individuals we used a Panasonic NV-GS250 camcorder (Panasonic Corporation, Kadoma, Japan). For better relating the acoustics to vocal anatomy, we selected for acoustic analyses the latest in the life recordings of the subject dholes. We took calls from 3–7 (mean 5.5 ± 1.9) separate recording sessions, 2.1–2.2 h per animal, 8.7 h of recordings in total. Only calls with high call-to-noise ratios, non-overlapped by background noise or calls of other individuals, non-disrupted by wind, and clearly identified as belonging to focal individuals were included in the analysis. We analyzed a total of 291 dhole calls (104 yaps, 89 biphonic yap-squeaks, and 98 squeaks) using Avisoft SASLab Pro software (Avisoft Bioacoustics, Berlin, Germany) with the Hamming window, FFT length 1024 points, frame 50% and overlap 93.75%.

We measured 5 (4 frequency, 1 temporal) acoustic variables per yap or squeak and 11 acoustic variables per biphonic yap-squeak (Table 1). On the screen in the spectrogram window we measured the duration with the standard marker cursor and the f0 and g0 variables with a free reticule cursor. The peak frequency was measured from the mean power spectrum. For the biphonic (yap-squeak) calls, peak frequencies of the yap and squeak components were measured after alternate 5 kHz high-pass and low-pass filtering. All measurements were exported automatically to Microsoft Excel (Microsoft Corp., Redmond, WA, USA). The acoustic measurements are presented in S1 Table.

**Table 1:** Measured acoustic variables and corresponding abbreviations.

Call variable	Low-frequency component	High-frequency component
Peak frequency (frequency with the maximum amplitude in the power spectrum) (kHz)	f peak	g peak
Maximum fundamental frequency (kHz)	f0 max	g0 max
Start fundamental frequency (kHz)	f0 start	g0 start
End fundamental frequency (kHz)	f0 end	g0 end
Duration of a component (ms)	f duration	g duration
Total duration of a biphonic call (ms)	biphonic call duration	

### Statistical analyses

Statistical analyses were conducted using STATISTICA, v. 6.0 (StatSoft, Tulsa, OK, USA). Means are presented as mean ± SD, all tests were two-tailed and differences were considered significant whenever *p* < 0.05. Only 16 of 80 distributions of measured parameter values did depart from normality (Kolmogorov-Smirnov test, *p* > 0.05). We used a two-way ANOVA with control of individual identity to compare the acoustics of the low-frequency component in dhole yaps or yap-squeaks with the high-frequency component in dhole squeaks or yap-squeaks.

## Results

### Dhole anatomy

**Fig 4:**
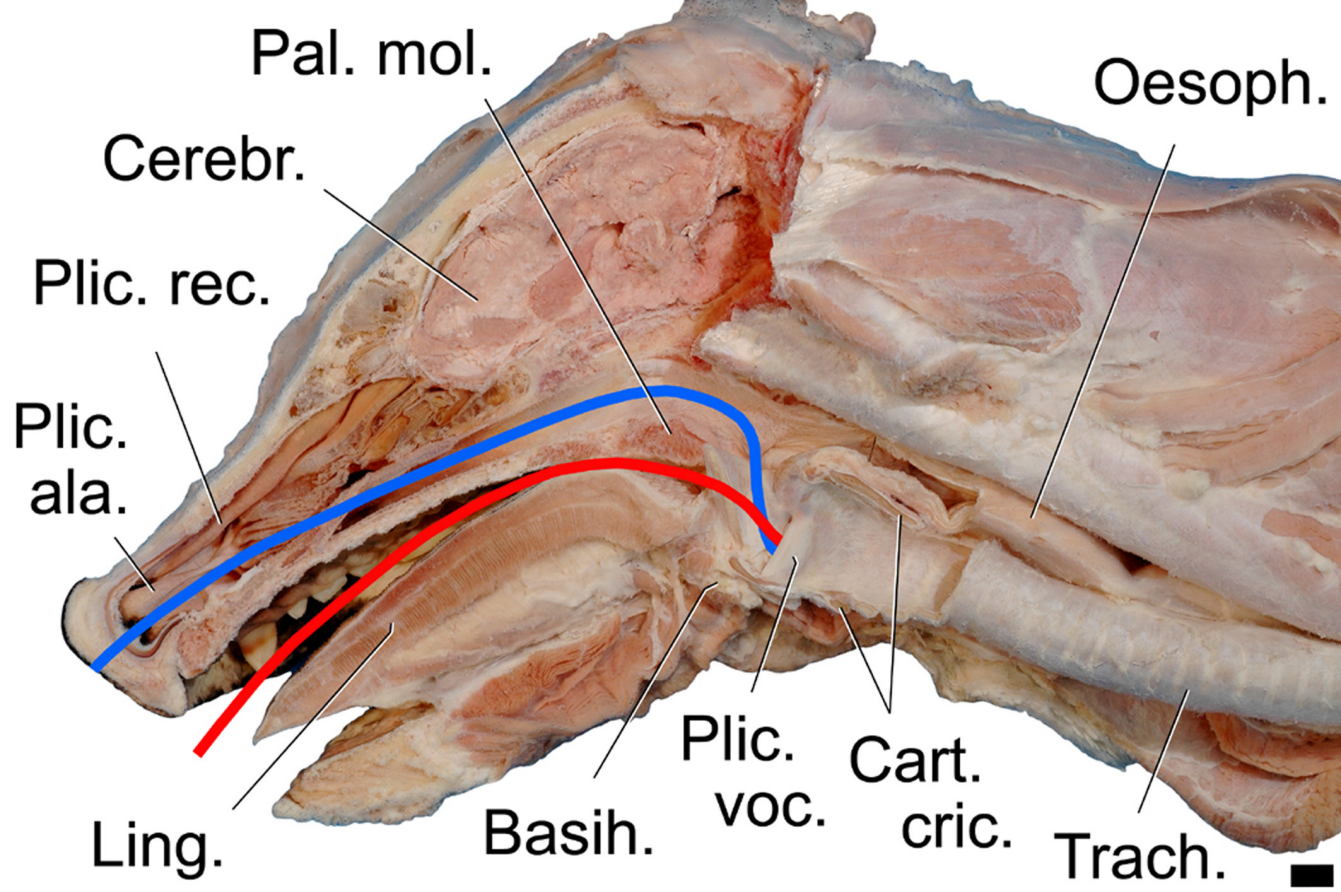
Mediosagittal section of the head and the region of the larynx of an adult male dhole. Illustrating the difference between nasal and oral vocal tract (vt) length including respective topographical relationships. The nasal vt is constantly longer than the oral vt in both adult male and adult female. The laryngeal entrance is depicted in a position for a nasal call. For production of an oral call, the soft palate is raised, the larynx slightly retracted and the epiglottis pulled ventrally to a position close to the root of the tongue, i.e. below the red line. Scale bar = 10 mm.

**Fig 5:**
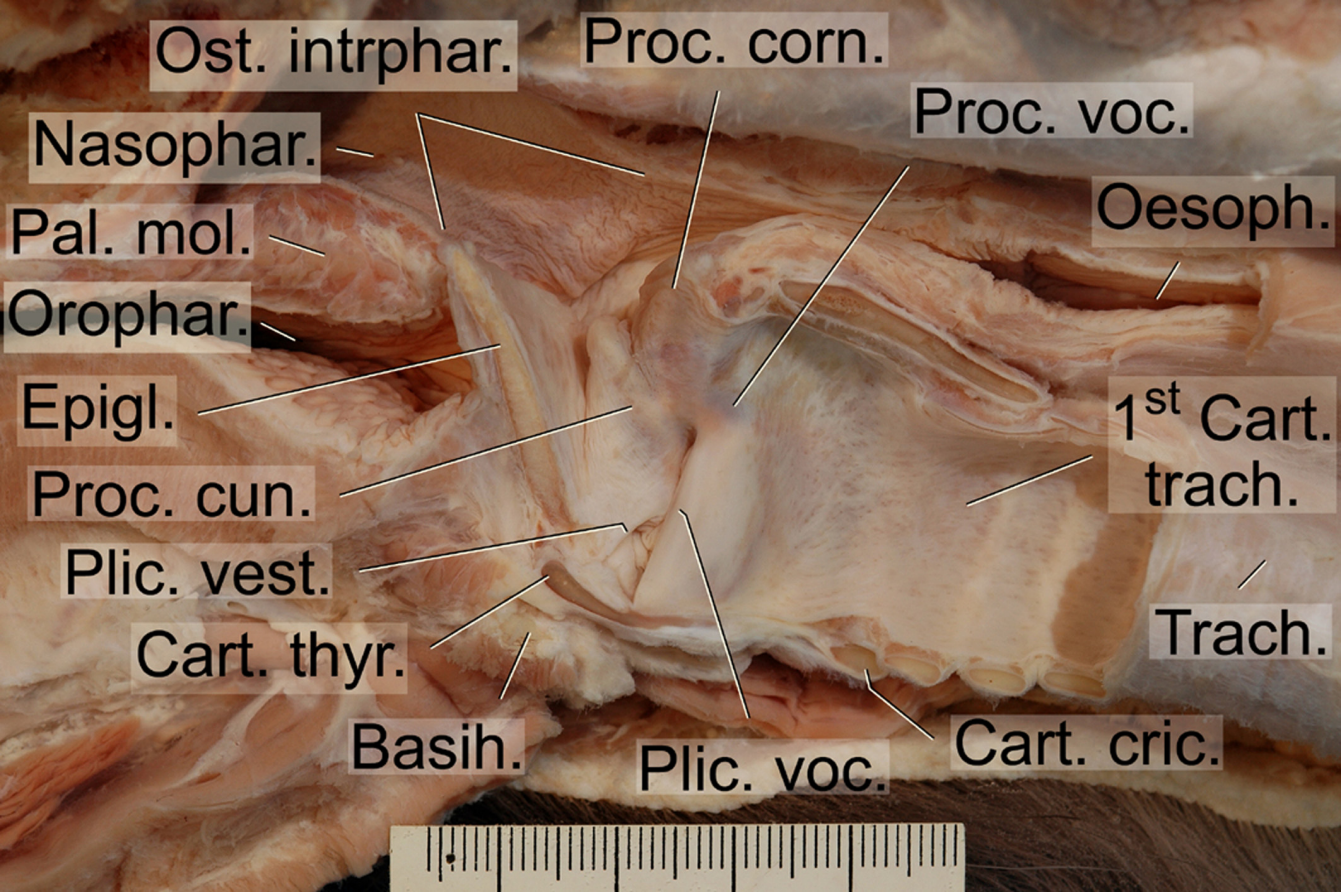
Intra-pharyngeal ostium in the dhole. During quiet respiration (not panting) and nasal call production the laryngeal entrance protrudes through the intra-pharyngeal ostium into the nasal portion of the pharynx. The intra-pharyngeal ostium is the sole connection between the ventral oral portion and the dorsal nasal portion of the pharynx. Mediosagittal section of pharynx and larynx of an unpreserved specimen, right half, medial view. Scale = 50 mm.

The overall morphology of the dhole vocal apparatus (Fig 4) revealed a larynx position immediately adjacent to the root of the tongue and the laryngeal entrance protruding through the intra-pharyngeal ostium into the nasopharynx. In this ‘respiratory position’ the epiglottis was overlapping the soft palate dorsally, i.e. it was located in a so-called ‘intra-narial position’ (Fig 5). No pronounced gender differences of the vocal apparatus were detected.

#### Vocal tract (vt)

The oral vt began with the laryngeal vestibulum and the oropharynx and then proceeded through the fauces to the oral cavity between the soft and hard palate dorsally and the tongue surface ventrally. The oral vt further proceeded between the teeth of the upper and lower jaw, through the oral vestibulum and ended at the oral opening (Fig 4). The dorsoventral dimensions of the oral vt depended on the gape. In the closed mouth situation it was only a 1–2 mm wide slit-like space, whereas in the open mouth situation it could be quite large depending on how much the dhole lower jaw is depressed. The oral vt length was 172 mm in the adult male and 162 mm in the adult female. The length of the trachea in the adult male, from the caudal edge of the cricoid cartilage to the bifurcation, amounted to 180 mm in the relaxed state and to 190 mm after moderate manual extension.

The nasal vt began with the laryngeal vestibulum and the intra-pharyngeal ostium of the soft palate connecting the ventral portions of the pharynx with the dorsal nasopharynx. The nasal vt continued rostrally between the base of the skull dorsally and the soft palate ventrally. After having passed the choanae, the nasal vt proceeded through the osseous nasal cavity along the ventral, common and middle nasal duct. During this passage its dorsoventral diameter expanded from about 7 mm at the choanae to about 17 mm at the ventral nasal concha and then decreased again to about 11 mm towards the nostril. In the flexible nostril region, the nasal vt coursed along the straight and alar fold before ending at the nasal opening (Fig 4). The nasal vt length, from the vocal folds up to the rostral edge of the nostrils was 208 mm in the adult male and 195 mm in the adult female.

#### Larynx

**Fig 6:**
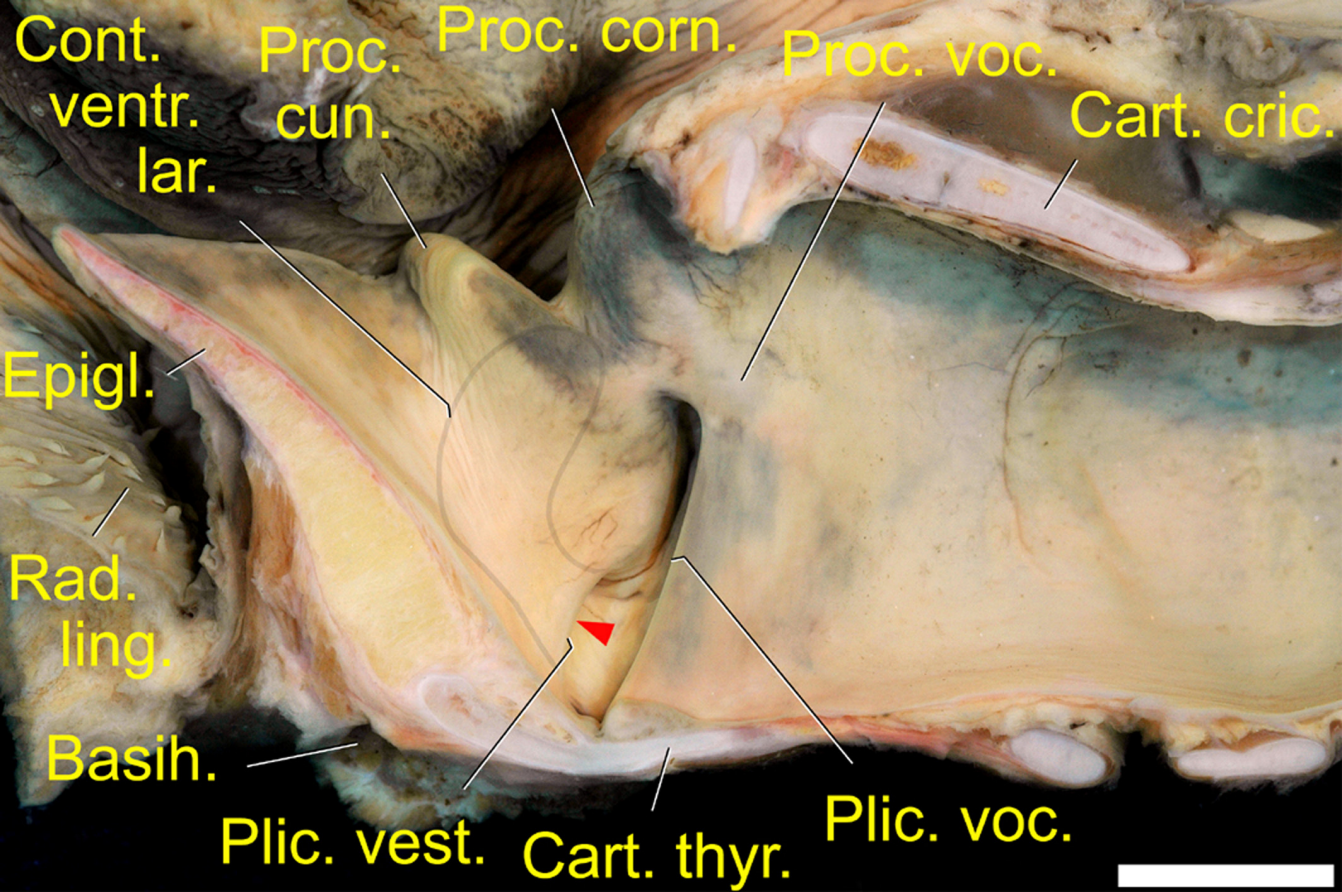
Mucous membrane relief of the larynx including the vocal and vestibular folds in the dhole. The contour of the lateral laryngeal ventricle is indicated. The position of the ventricle is rostral to the vestibular fold and lateral to the cuneiform process. The red arrow points into the caudally directed entrance to the lateral laryngeal ventricle. Mediosagittal section of the formalin-fixed larynx of a two-years-old female, right half, medial view. White scale bar = 10 mm.

The overall length of the larynx in the adult female, from the rostral tip of the epiglottis to the caudal edge of the cricoid cartilage was about 50 mm (53 mm in the adult female No 3 larynx); the overall dorsoventral height was about 30 mm (31 mm in the adult female No 3 larynx). The distance between the root of the tongue and the epiglottis was about 5 mm. The resting angle of the epiglottis relative to the longitudinal axis of the larynx was around 45°. The dhole larynx had a typical mammalian vocal fold with a free and flexible rostral portion ending in a sharp edge. This rostrally directed edge of the vocal fold was supported by a vocal ligament and laterally covered by the thyroarytenoid muscle. Vocal membranes, i.e. thin and flexible rostral extensions of the vocal folds, were not detected. The vocal fold extended between the vocal process of the arytenoid cartilage dorsally and the mid-dorsal surface of the thyroid cartilage ventrally. Dorsoventral length of the vocal fold was 16.4 mm in the adult male and 15.5 mm in the adult female. Its angle, relative to the longitudinal axis of the larynx, was around 115°. The length of the vocal ligament corresponded to the length of the vocal fold (Fig 6).

Rostral to the vocal fold, the dhole larynx had a lateral laryngeal ventricle (LLV) on each side. The LLV was accommodated by the lateral, concave surface of the cuneiform process. The sharp medial edge of its caudally directed opening made up the vestibular fold that was situated 4–5 mm rostral to the vocal fold. The vestibular fold extended between the ventral end of the cuneiform process dorsally and the mid-dorsal surface of the thyroid cartilage ventrally, 1–2 mm rostral to the ventral attachment of the vocal fold. The caudally directed edge of the vestibular fold was supported by a vestibular ligament of corresponding length. Dorsoventral length of the vestibular fold was 6.7 mm in the adult male and 7 mm in the adult female. Its angle, relative to the longitudinal axis of the larynx, was almost the same as that of the vocal fold. However, in contrast to the latter, its sharp edge was directed caudally (Fig 6). The resting dimensions of the right lateral laryngeal ventricle were 9.85 mm in dorsoventral height and 6.0 mm in rostrocaudal length in the adult male. The vestibular fold occupied a slightly more medial position (about 1 mm) than the vocal fold. The major portion of the LLV, except its caudalmost narrow ‘neck’, was not covered laterally by the uniform thyroarytenoid muscle (S2 Table).

#### Laryngeal cartilages

**Fig 7:**
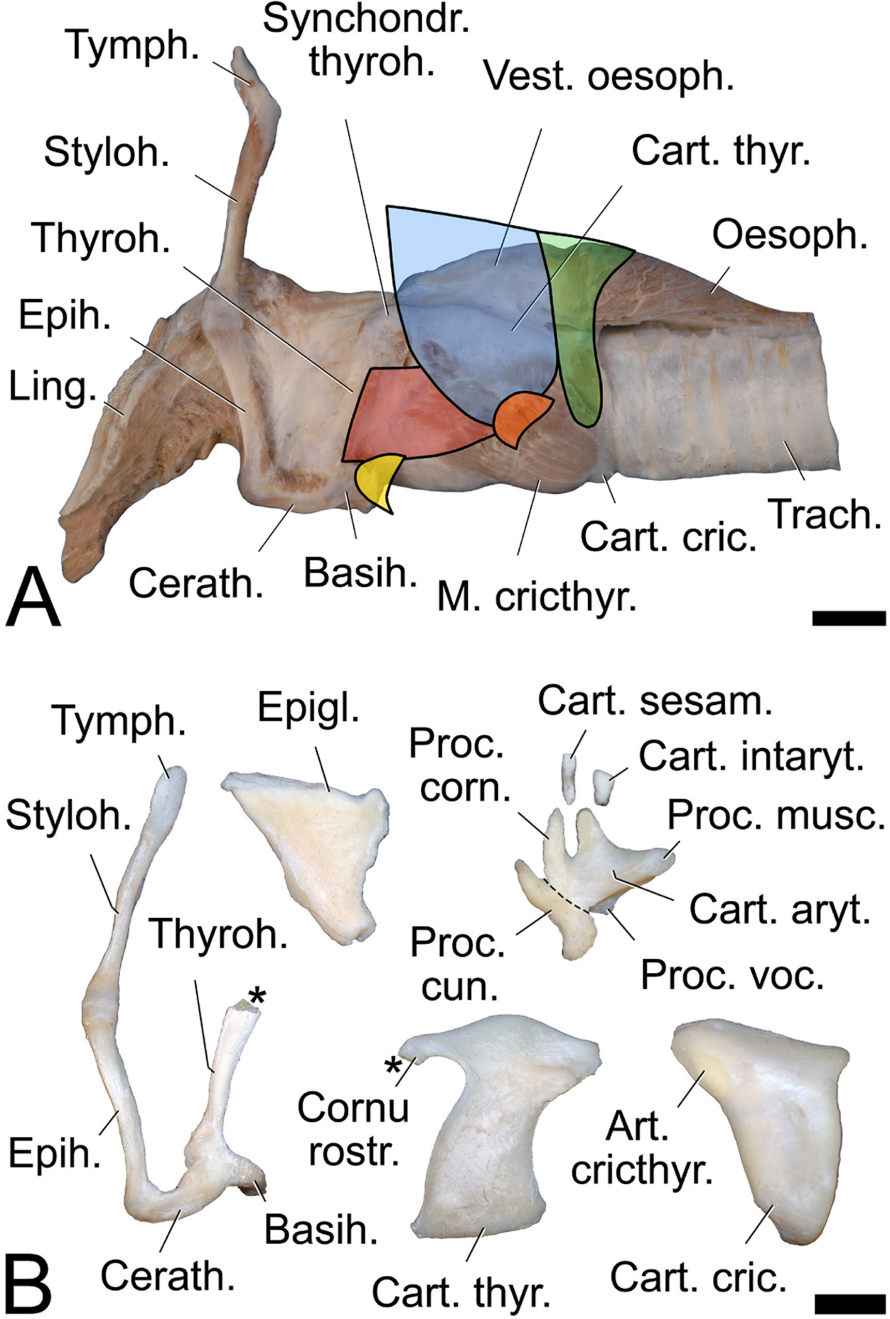
Hyoid apparatus (A) and laryngeal cartilages (B) of the dhole. (A) Excised hyoid apparatus and larynx; overlying muscles partly reconstructed on the basis of macroscopic dissection. The thyrohyoid 'articulation' is established by a cartilaginous connection. (B) Left half of hyoid apparatus and laryngeal cartilages. The interarytenoid cartilage is intercalated between left and right arytenoid cartilage. A sesamoid cartilage supports the transverse arytenoid muscles at their dorsomedian fusion along the transverse furrow between the corniculate and medial processes of the arytenoid cartilages. Colours in (A): green = M. cricopharyngeus; blue = M. thyropharyngeus; red = M. thyrohyoideus; orange = termination of M. sternothyroideus; yellow = termination of M. sternohyoideus. ** in (B): thyrohyoid connection. (A) and (B): Left lateral view. Scale bar = 10 mm, respectively.

The dhole larynx comprised 9 cartilages when counting the paired cuneiform processes as separate cartilages. However, two of the cartilages were very small. Rostrally, there was the unpaired epiglottis, the lateral process of which was connected to the cuneiform process within the aryepiglottic fold. The paired cuneiform processes, about mid-way of their caudal contour, were punctually connected to the rostral edge of the arytenoid cartilages. Therefore, the cuneiform “process” was more like a separate cartilage than a mere appendage of the arytenoid cartilage. Both the dorsal branch of the cuneiform process and the corniculate process of the arytenoid cartilage were set against the longitudinal axis of the larynx at the angle of about 45°. The ventral branch of the cuneiform process was positioned parallel to the rostral edge of the vocal fold, its ventral knob-like end serving as a dorsal attachment point for the vestibular fold (Figs 6 and 7). The paired arytenoid cartilages had a deep incisure dorsally, between the corniculate process and the medial process. In this incisure, the small, rod-like sesamoid cartilage was embedded in the two transverse arytenoid muscles where they met in the dorsal midline. Caudally adjacent, the small, trapezoid-shaped, interarytenoid cartilage was intercalated between the medial processes of the left and right arytenoid cartilages. The region of the LLV, including the vestibular and vocal folds, was ventrally and laterally covered by the unpaired thyroid cartilage. Its rostral horns established the short (about 4 mm) cartilaginous connection to the thyrohyoid of the hyoid apparatus. Its caudal horns were connected *via* the cricothyroid articulations to the cricoid cartilage. Dorso- and ventrolateral faces of the thyroid lamina were separated by the oblique line which thickened caudally into a distinct knob. Caudally, followed the unpaired cricoid cartilage that articulated with the two arytenoid cartilages at its rostrolateral edges *via* the paired cricoarytenoid articulation. Caudolaterally, the cricoid cartilage connected to the caudal horns of the thyroid cartilage *via* the paired cricothyroid articulation (Fig 7).

#### Hyoid apparatus

The approximate resting position of the hyoid apparatus is depicted in Fig 7. The hyoid apparatus of the dhole consisted of the 11 parts typical for mammals: paired tympanohyoids, stylohyoids, epihyoids and ceratohyoids that suspended the hyoid apparatus from the skull and connected it to the unpaired, transversely oriented, basihyoid immediately ventral to the base of the epiglottis. The basihyoid was located ventrally adjacent to where the base of the epiglottis was fused to the rostral edge of the thyroid cartilage. Dorsally attached to the basihyoid were the paired thyrohyoids, whose caudal ends were connected to the rostral horns of the thyroid cartilage *via* a piece of cartilage, instead of a true thyrohyoid articulation typical for most mammals (Fig 7). Along their caudal edge, the thyrohyoids were connected to the oblique rostral edge of the thyroid cartilage by the short thyrohyoid membrane.

#### Intrinsic laryngeal muscles

Origins and insertions of the intrinsic laryngeal muscles including their respective functions are listed in S2 Table. Functions are given after [30], modified under consideration of [45]. The fixation of the thyroid cartilage, that is necessary for the coordinated movement of the remaining laryngeal cartilages, is established by joint action of the sternothyroid and thyrohyoid muscles together with other hyoid muscles.

**Fig 8:**
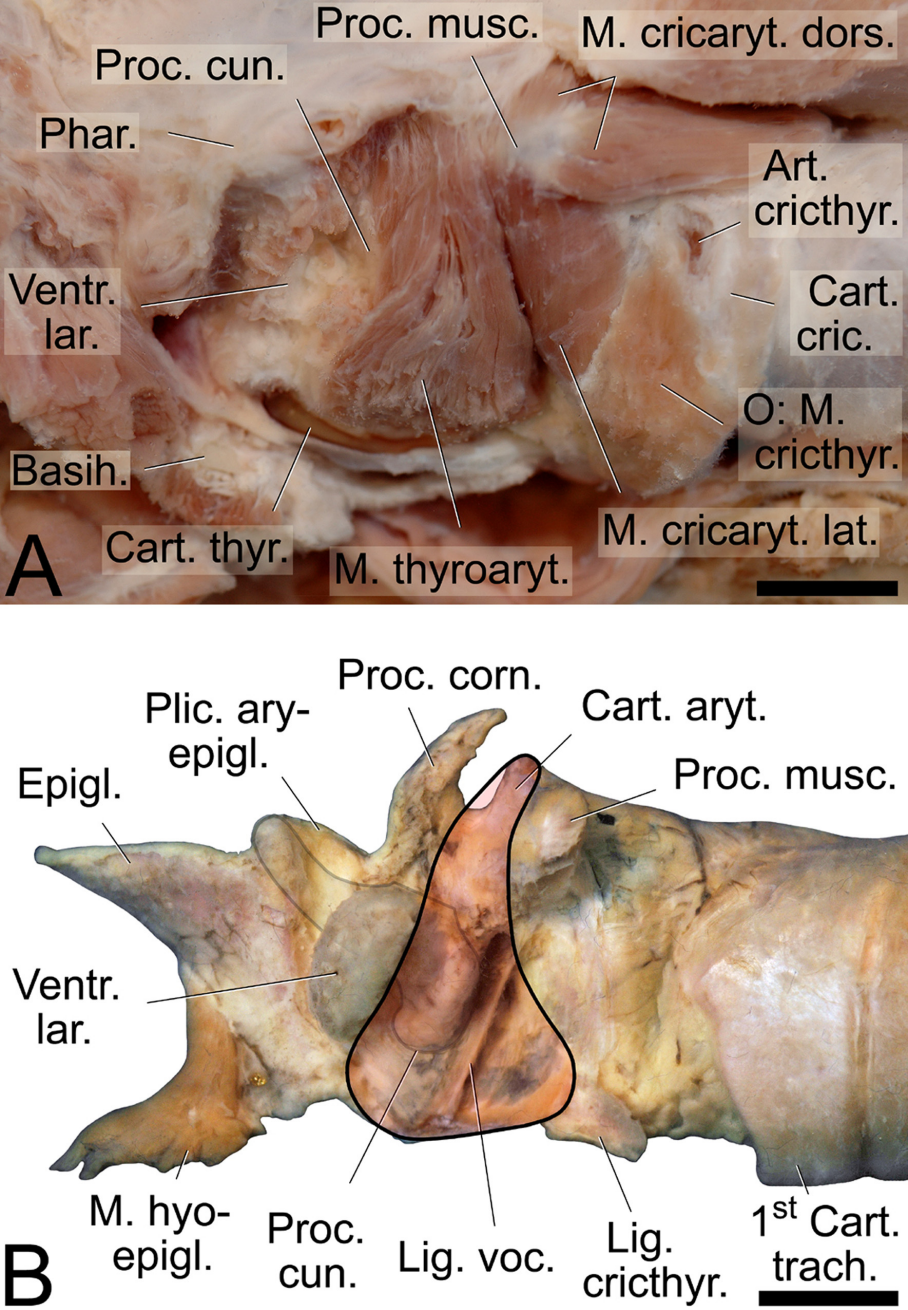
Intrinsic laryngeal muscles of the dhole (A) and position of lateral laryngeal ventricle (B). (A) Larynx after removal of the left cricothyroid muscle and the left half of the thyroid cartilage. Dorsal and lateral cricoarytenoid muscles and thyroarytenoid muscle exposed. The thyroarytenoid muscle is not divided into a rostral ventricularis and a caudal vocalis muscle. (B) Larynx after removal of its intrinsic muscles. Reconstruction of the thyroarytenoid muscle (black line, shaded in translucent red) shows how its wider ventral portion covers the ventral end of the cuneiform process and the ‘neck’ of the laryngeal ventricle rostrally. Contour of the cuneiform process indicated by faint black line. The vocal ligament marks position and angle of the medially located vocal fold. (A) and (B): Left lateral view. Scale bar = 10 mm.

M. cricothyroideus originated from the lateroventral half of the cricoid arch and terminated on the caudolateral part of the thyroid lamina, ventrally adjacent to the caudal knob of the oblique line. Its border of termination ran in an oblique manner from caudodorsally, adjacent to the caudal horn of the thyroid cartilage, to rostroventrally. Caudally, its termination was covered by the sternothyroid muscle and rostrally, by the thyrohyoid muscle which terminated on and originated from the caudal knob of the oblique line of the thyroid cartilage, respectively (Figs 2 and 7B and 8A).

M. cricoarytenoideus dorsalis originated from the ipsilateral half of the cricoid lamina and terminated on the caudomedial and caudolateral aspects of the muscular process of the arytenoid cartilage (Fig 8A). M. cricoarytenoideus lateralis was the short muscle that originated laterorostrally from the edge of the cricoid arch and terminated on the ventral aspect of the muscular process of the arytenoid cartilage (Fig 8A).

M. arytenoideus transversus originated from the rostromedial aspect of the muscular process of the arytenoid cartilage and from the arcuate line. It coursed dorsomedially and, in the median plane and rostral to the interarytenoid cartilage, terminated on its contra-lateral counterpart supported by the sesamoid cartilage, respectively.

M. thyroarytenoideus was not divided into a ventricularis and a vocalis muscle. Instead, it originated uniformly from the caudal half of the dorsal surface of the thyroid cartilage plus the cricothyroid ligament along a paramedian line. Tapering pronouncedly to about one fourth of its ventralmost diameter, its fibers coursed dorsally to terminate on the vocal process, the arcuate line of the arytenoid cartilage and on the rostral face of the transverse arytenoid muscle (Fig 8A and 8B). In its middle part, this muscle covered the ventral end of the cuneiform process and its rostroventral portion covered the ‘neck’ of the laryngeal ventricle laterally, i.e. the caudally directed opening and its initial, rostrally directed, narrow portion (Fig 8B).

#### Extrinsic laryngeal muscles, hyoid muscles and muscles of the fauces

**Fig 9:**
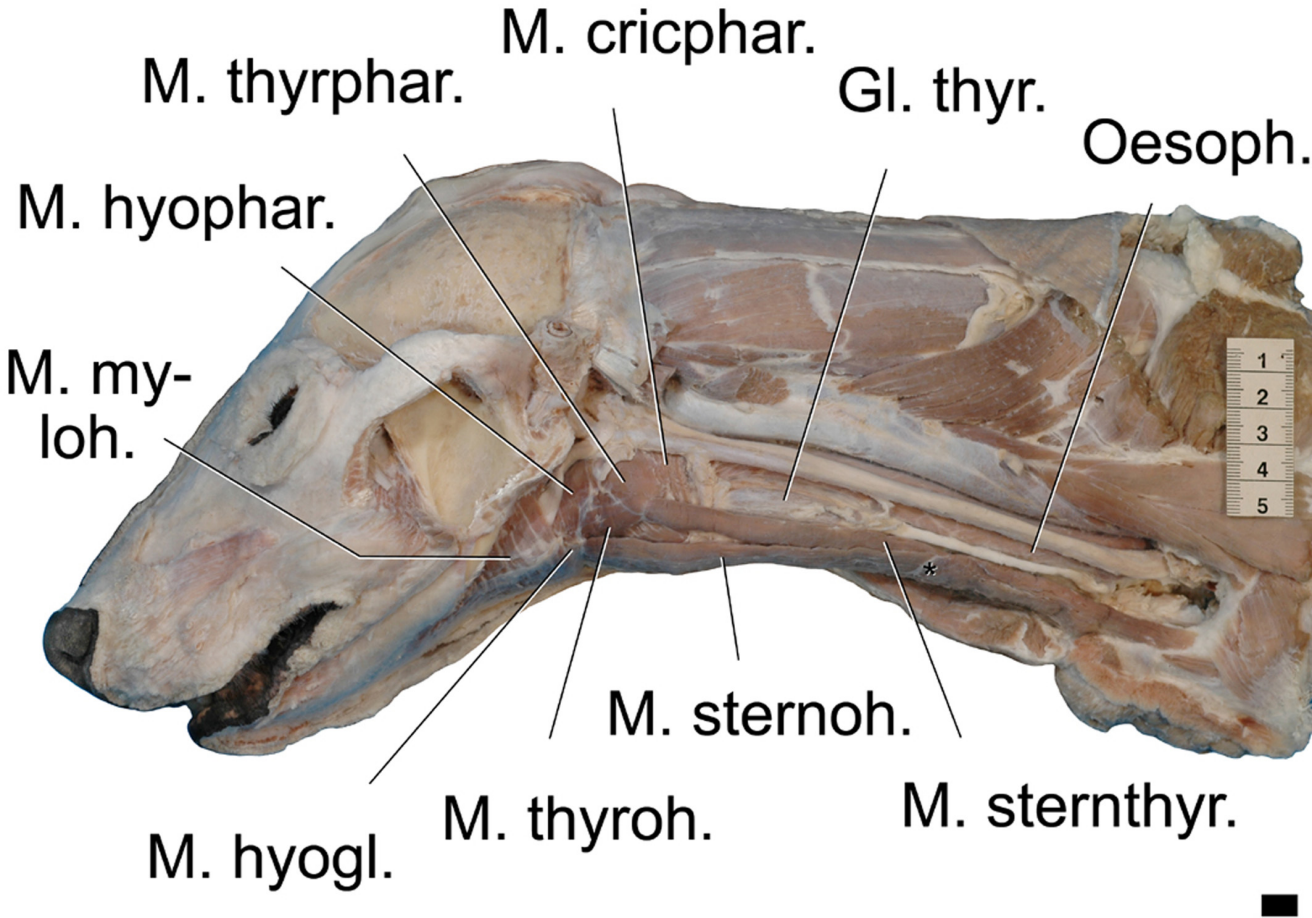
Extrinsic laryngeal muscles and ventral hyoid muscles of the dhole. The sternohyoid and sternothyroid muscles are fused between their joint origin from the sternal manubrium up to a tendinous inscription. At this point both muscles separate to reach their terminations on the basihyoid and on the thyroid cartilage, respectively. * = position of tendinous inscription. Left lateral view. Scale = 50 mm.

Origins and insertions of these muscles including their respective functions are listed in S3 Table and Fig 9. Functions are given after [30].

#### Organs of the nasal vocal tract

**Fig 10:**
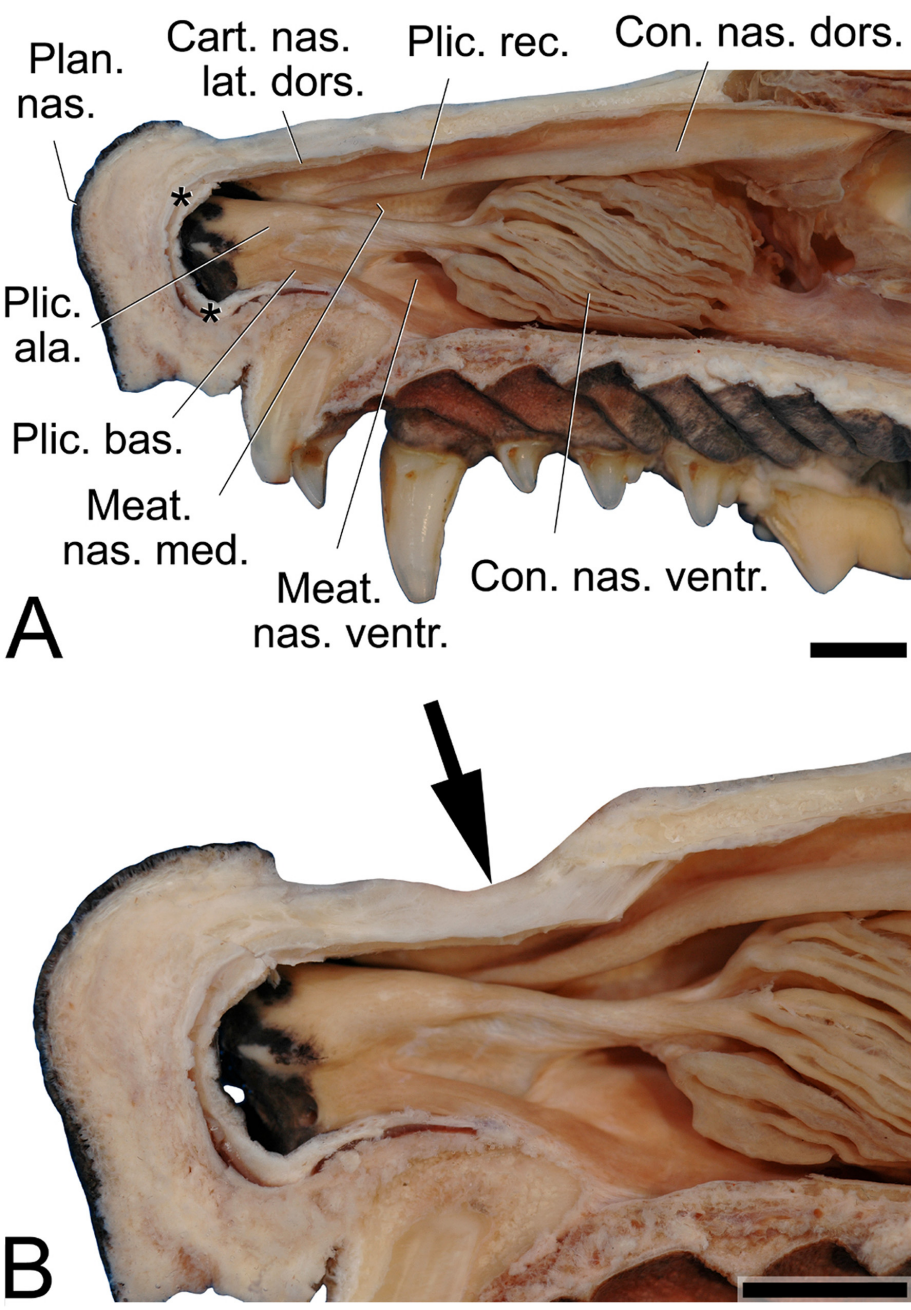
Right nasal cavity (A) and its flexible rostral portion (B) of an adult male dhole. (A) Middle nasal concha and nasal septum removed to expose the dorsal, the alar and the basal folds. ** = cut edge of the nasal septum. (B) Detail of (A). The arrow points to the flexible rostral portion of the nose, which can be variably constricted by differential action of the rostral nasal muscles. This will narrow particularly the space between the dorsal and the alar folds and, in concert with movements of the nostril wings, might influence nasal call characteristics. Mediosagittal section of the nasal region, right half, medial view. Scale bar = 10 mm in (A) and (B), respectively.

Structures inside the nasal cavity were inspected, checked for flexible elements and the dorsoventral and transverse diameters of the nasal vt were measured. In the caudal three quarters of the nasal cavity, the thin mucous membrane surfaces of the ethmoturbinals and of the dorsal, middle and ventral nasal conchae were all supported by osseous lamellae and, therefore, can be considered as being inflexible relative to a passing airstream. However, rostral to the ventral nasal concha and the rostral tip of the nasal bone, i.e. in the nostril region, structures were supported by cartilage and connective tissue and, therefore, possessed a certain extent of flexibility. Major relevant structures were the straight fold (dorsally), the alar fold (intermediate) and the basal fold (ventrally) (Fig 10).

#### Red fox vocal organs and hyoid apparatus

**Fig 11:**
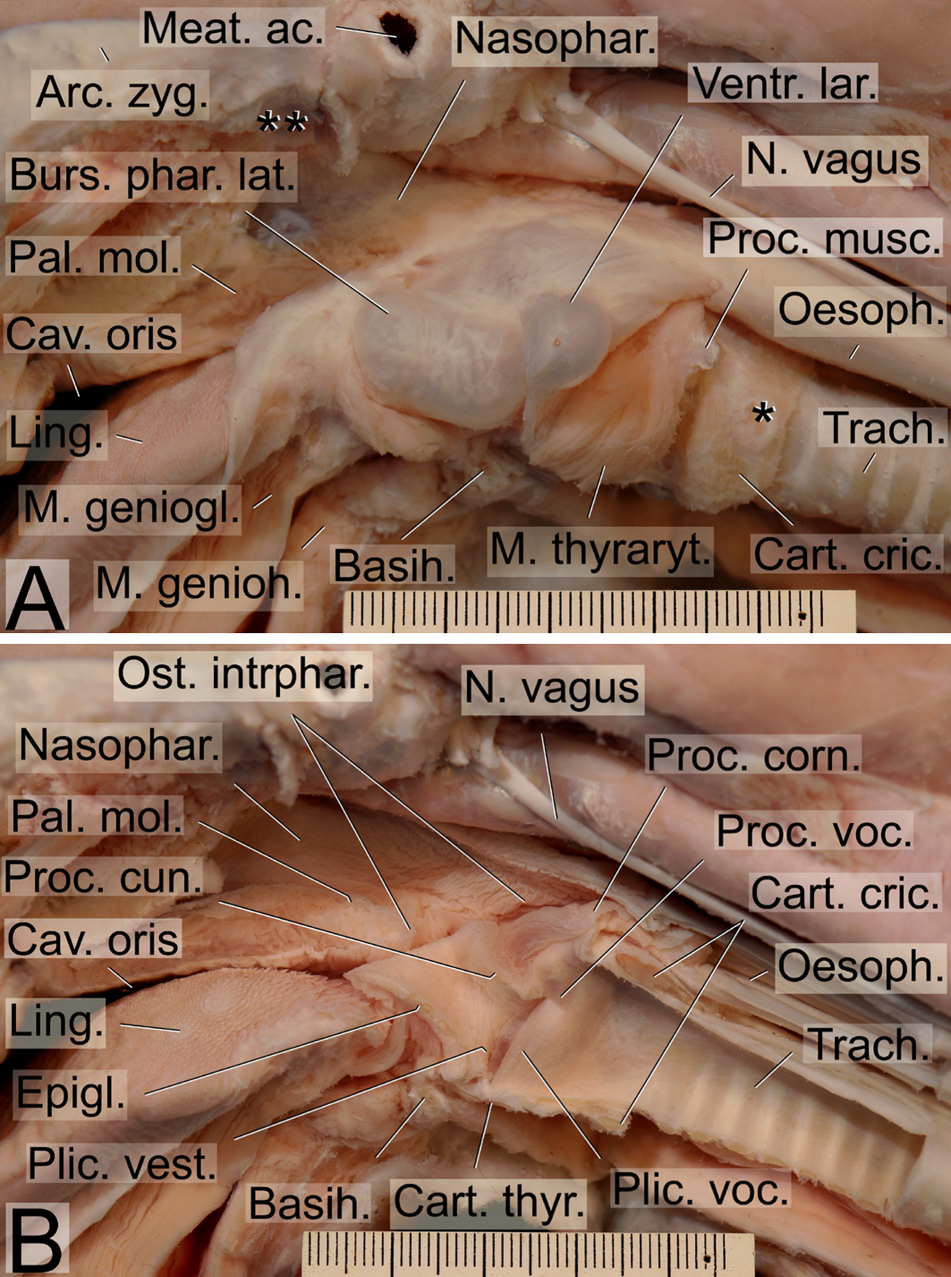
Lateral laryngeal ventricle (A) and larynx (B) in red fox. (A) Artificially inflated pharynx and laryngeal ventricle after removal of the left half of the thyroid cartilage. The thyroarytenoid muscle is not divided into a rostral ventricularis and a caudal vocalis muscle. Its wider ventral portion covers the ‘neck’ of the laryngeal ventricle rostrally. (B) Mucous membrane relief of the larynx including the vocal and vestibular folds in the red fox. In (A): * = cricothyroid articulation; ** = temporomandibular articulation. Left lateral view; in (B): Mediosagittal section of pharynx and larynx of an unpreserved specimen, right half, medial view. Scale = 50 mm in (A) and (B), respectively.

In both sexes of red fox the larynx was located immediately adjacent to the root of the tongue. In the resting position, the laryngeal entrance protruded through the intra-pharyngeal ostium into the nasopharynx (Fig 11). In the adult male, the oral vt length was 143 mm and the nasal vt length was 167 mm. In the region of the piriform recess, the oropharynx was expandable as sort of bilateral pharyngeal pouch (Fig 11A). Rostral to the vocal fold, there was a lateral laryngeal ventricle whose opening was medially covered by the vestibular fold. The sharp edge of the vestibular fold was directed caudally. The LLV extended rostral to the vestibular fold. Its narrow ‘neck’ portion is covered by rostroventral parts of the thyroarytenoid muscle. The vocal fold had a free and flexible rostral portion ending in a sharp, rostrally directed edge. In the adult male, vestibular fold length was 4.2 mm and vocal fold length was 11.5 mm. The thyroarytenoid muscle was uniform and not divided into a ventricularis and a vocalis muscle; its fibres converged considerably from ventral origin to dorsal termination (Fig 11A).

When considering the cuneiform processes as separate cartilages, the red fox possesses 7 major laryngeal cartilages: epiglottis, thyroid cartilage, paired cuneiform processes, paired arytenoid cartilages, cricoid cartilage. Potential interarytenoid and sesamoid cartilages were not investigated. The thyrohyoid connection was cartilaginous. The hyoid apparatus consisted of the 11 parts typical for carnivores [46].

#### Acoustics of live dholes

During their life time, the 4 dholes, which served as anatomical specimens for this study, produced all the three types of contact calls: the low-frequency yaps, the high-frequency squeaks and the biphonic yap-squeaks (Table 2). A comparison of the low-frequency component variables between the yap and yap-squeak call types showed that the onset values of f0 and f0 max were significantly higher in the yap-squeaks than in the yaps (Table 3). The values of all other variables were undistinguishable between these two call types. At the same time, we found a significant effect of individual identity on all variables of the low-frequency component of dhole contact calls (Table 3). Similarly, a comparison of the high-frequency component variables between the squeak and yap-squeak call types showed that only the onset values of g0 were significantly higher in the yap-squeak than in the squeak (Table 4). The values of all other variables were undistinguishable between these two call types. As for the low-frequency component, we found a significant effect of individual identity on all variables of the high-frequency component of dhole contact calls (Table 4).

**Table 2:** Values (mean ± SD) of acoustic variables measured in calls of 4 dholes which served postmortem as anatomical specimens for this study.

Call variable	Male 1 (Mun)	Female 2 (Nika)	Female 3 (Vika)	Female 4 (Svet)	All animals
Yaps, n =	47	3	9	45	104
f peak, kHz	0.75 ± 0.31	1.43 ± 1.11	1.20 ± 0.67	1.43 ± 0.92	1.10 ± 0.76
f0 max, kHz	0.82 ± 0.07	0.91 ± 0.05	0.97 ± 0.08	0.94 ± 0.06	0.89 ± 0.09
f0 start, kHz	0.67 ± 0.11	0.79 ± 0.05	0.93 ± 0.15	0.81 ± 0.08	0.75 ± 0.13
f0 end, kHz	0.57 ± 0.09	0.77 ± 0.10	0.70 ± 0.07	0.71 ± 0.12	0.65 ± 0.12
f duration, ms	176 ± 142	81 ± 28	43 ± 10	85 ± 18	122 ± 108
Squeaks, n =	4	37	39	18	98
g peak, kHz	9.42 ± 1.36	9.47 ± 0.41	8.46 ± 0.54	8.85 ± 0.84	8.95 ± 0.75
g0 max, kHz	10.24 ± 0.67	9.83 ± 0.44	8.75 ± 0.72	9.28 ± 0.57	9.32 ± 0.78
g0 start, kHz	8.46 ± 0.72	8.97 ± 0.70	6.67 ± 1.17	7.27 ± 1.20	7.72 ± 1.45
g0 end, kHz	9.52 ± 0.27	9.68 ± 0.40	8.31 ± 0.75	8.34 ± 1.20	8.88 ± 0.99
g duration, ms	122 ± 48	135 ± 26	89 ± 16	119 ± 42	113 ± 34
Yap-squeaks, n =	7	32	25	25	89
g peak, kHz	9.55 ± 0.82	9.36 ± 0.21	8.57 ± 0.39	8.90 ± 1.17	9.02 ± 0.78
g0 max, kHz	9.80 ± 0.80	10.00 ± 0.23	8.74 ± 0.43	9.21 ± 1.12	9.41 ± 0.86
g0 start, kHz	8.92 ± 0.56	9.63 ± 0.52	6.56 ± 0.56	8.78 ± 1.10	8.47 ± 1.45
g0 end, kHz	8.81 ± 1.02	9.65 ± 0.22	8.49 ± 0.47	8.24 ± 1.73	8.86 ± 1.16
g duration, ms	100 ± 23	142 ± 23	105 ± 20	92 ± 35	114 ± 34
f peak, kHz	1.34 ± 1.10	0.93 ± 0.09	1.20 ± 0.54	1.24 ± 0.71	1.13 ± 0.57
f0 max, kHz	0.94 ± 0.27	1.05 ± 0.07	1.02 ± 0.12	0.98 ± 0.11	1.01 ± 0.12
f0 start, kHz	0.83 ± 0.33	0.93 ± 0.11	0.95 ± 0.10	0.80 ± 0.10	0.89 ± 0.15
f0 end, kHz	0.78 ± 0.22	0.60 ± 0.13	0.73 ± 0.14	0.71 ± 0.13	0.68 ± 0.15
f duration, ms	81 ± 40	90 ± 34	47 ± 14	92 ± 39	78 ± 37
biphonic call duration, ms	111 ± 27	152 ± 25	110 ± 18	116 ± 39	127 ± 34

Designations: Yap = the low-frequency call; Squeak = the high-frequency call; Yap-squeak = the biphonic call.

**Table 3:** Controlled for individual identity two-way ANOVA results for comparison of low-frequency component variables between the yap and yap-squeak call types in the dhole.

Call variable	Factor	
	Call type	Individual identity
f peak	*F*_*1*,*188*_ = 0.05; *P* = 0.83	***F***_***3*,*188***_ = **7.95; *P* < 0.001**
f0 max	***F***_***1*,*188***_ = **13.26; *P* < 0.001**	***F***_***3*,*188***_ = **18.42; *P* < 0.001**
f0 start	***F***_***1*,*188***_ = **4.81; *P* < 0.05**	***F***_***3*,*188***_ = **22.75; *P* < 0.001**
f0 end	*F*_*1*,*188*_ = 2.13; *P* = 0.15	***F***_***3*,*188***_ = **11.48; *P* < 0.001**
f duration	*F*_*1*,*188*_ = 0.94; *P* = 0.33	***F***_***3*,*188***_ = **14.14; *P* < 0.001**

*F*: *F*-ratios of two-way ANOVA. Significant effects are presented in bold.

**Table 4:** Controlled for individual identity two-way ANOVA results for comparison of high-frequency component variables between the squeak and yap-squeak call types in the dhole.

Call variable	Factor	
	Call type	Individual identity
g peak	*F*_*1*,*182*_ = 0.02; *P* = 0.89	***F***_***3*,*182***_ = **24.51; *P* < 0.001**
g0 max	*F*_*1*,*182*_ = 0.06; *P* = 0.81	***F***_***3*,*182***_ = **40.14; *P* < 0.001**
g0 start	***F***_***1*,*182***_ = **17.84; *P* < 0.001**	***F***_***3*,*182***_ = **86.21; *P* < 0.001**
g0 end	*F*_*1*,*182*_ = 0.01; *P* = 0.93	***F***_***3*,*182***_ = **32.55; *P* < 0.001**
g duration	*F*_*1*,*182*_ = 0.03; *P* = 0.86	***F***_***3*,*182***_ = **29.06; *P* < 0.001**

*F*: *F*-ratios of two-way ANOVA. Significant effects are presented in bold.

Video analysis and direct observations of calling dholes and domestic dogs revealed that squeaks are produced with their mouth closed. In contrast, yaps of dholes and whines of domestic dogs were produced with their mouth open. When dholes or dogs shifted audibly from the high to the low frequency, a slight opening of the mouth could be observed. Several squeaks of individual dholes and dogs were accompanied by a tensioning of the wings of the nose. During the dhole squeaks, we observed an additional tensioning of ventral neck muscles in the area of the larynx.

Discussion

The laryngeal morphologies of the dhole and red fox lack conspicuous differences. At least, our comparison failed to reveal any specific anatomical adaptations of the dhole larynx to the production of high-frequency and biphonic vocalizations. There are two possible explanations for this result: 1) a typical canid larynx is capable of producing the low-frequency calls, high-frequency calls and biphonic calls without particular anatomical adaptations or 2) the presence or the lack of high-frequency and biphonic components in the acoustic repertoire appears to depend on other, non-laryngeal features. Below we discuss potential mechanisms for production of g0 and biphonations in the dhole based of main anatomical findings of this study. We also connect our findings with published data on auditory and social differences between dog-like and fox-like canids.

### Auditory and social differences between dog-like and fox-like canids

We failed to detect conspicuous differences between the dhole and red fox laryngeal morphologies. However, only the dholes can produce the second fundamental frequency (g0) and biphonation. If red foxes are also physically capable of producing g0 and biphonation, why these components lack in their vocal repertoires? The answer can be found in different auditory capacities between the dog-like and fox-like canids. The overall frequency ranges for hearing are similar in dog-like canids and in fox-like canids. The high-frequency cut-off range is 41–47 kHz in the domestic dog, depending on the dog breed [47], whereas it is 48–51 kHz in red fox [48], 20 kHz in the kit fox [49] and 16 kHz in the Arctic fox [50]. However, the peak hearing sensitivity for the dog-like canids is shifted remarkably to higher frequencies. For instance, in dachshund dogs, the peak hearing sensitivity is at 8 kHz [47] and it corresponds well to the g0 values of 6.5–7 kHz in this dog pedigree (Fig 1A) [8,9]. At the same time, the peak hearing sensitivity of similar-sized red fox is substantially lower, at 2 kHz [51] or at 4 kHz [48]. Other species of fox-like canids display similarly low peak hearing sensitivities, e.g. 2–4 kHz in the kit fox *Vulpes macrotis* [49] and about 4 kHz in the Arctic fox [50]. Further support for the low-frequency peak of audition in red fox comes from the findings that the malleus of the auditory ossicles is heavier in red fox than in the dog [52]. A higher mass of the malleus is indicative of improved low-frequency hearing, as soon as the high-frequency hearing in air is constrained by the ossicle inertia [53–55].

The lower peak hearing sensitivity in red fox is probably related to their predation habits. With such hearing acute at lower frequencies, red foxes can detect their small rodent prey even when it is silent, based on the low-frequency noises resulting from the prey almost restless movements [56,57].

Pack hunting in the dhole [58–61] is associated with their obligate sociality [62–64], whereas hunting small muroid prey by fox-like canids does not need in cooperation with conspecifics [56,57]. Pack-living in the dhole needs in delicate social relationships [20,65,66] to relax the intra-pack aggression [58,62,63,66,67]. Dhole biphonic yap-squeaks better discriminate individuals compared to either yaps or squeaks alone [4] and better encode the position of a caller relative to a listener owing to different propagation of the low and high fundamental frequencies [68]. This allows easy and quick recognition of vocalizing individuals along to directionality of their approach. By contrast, red foxes are social only during the breeding season [69]. Consistently, the producing high percentages of biphonic calls African wild dogs are obligate social [1], whereas polar foxes, which are not known to produce biphonations, are only facultatively social [70,71]. Thus, selection pressures favoring production and perception of biphonations appears to be present in dog-like canids but lacking in fox-like canids.

### Potential sources for high-frequency and biphonic vocalization

#### Vortex shedding

**Fig 12:**
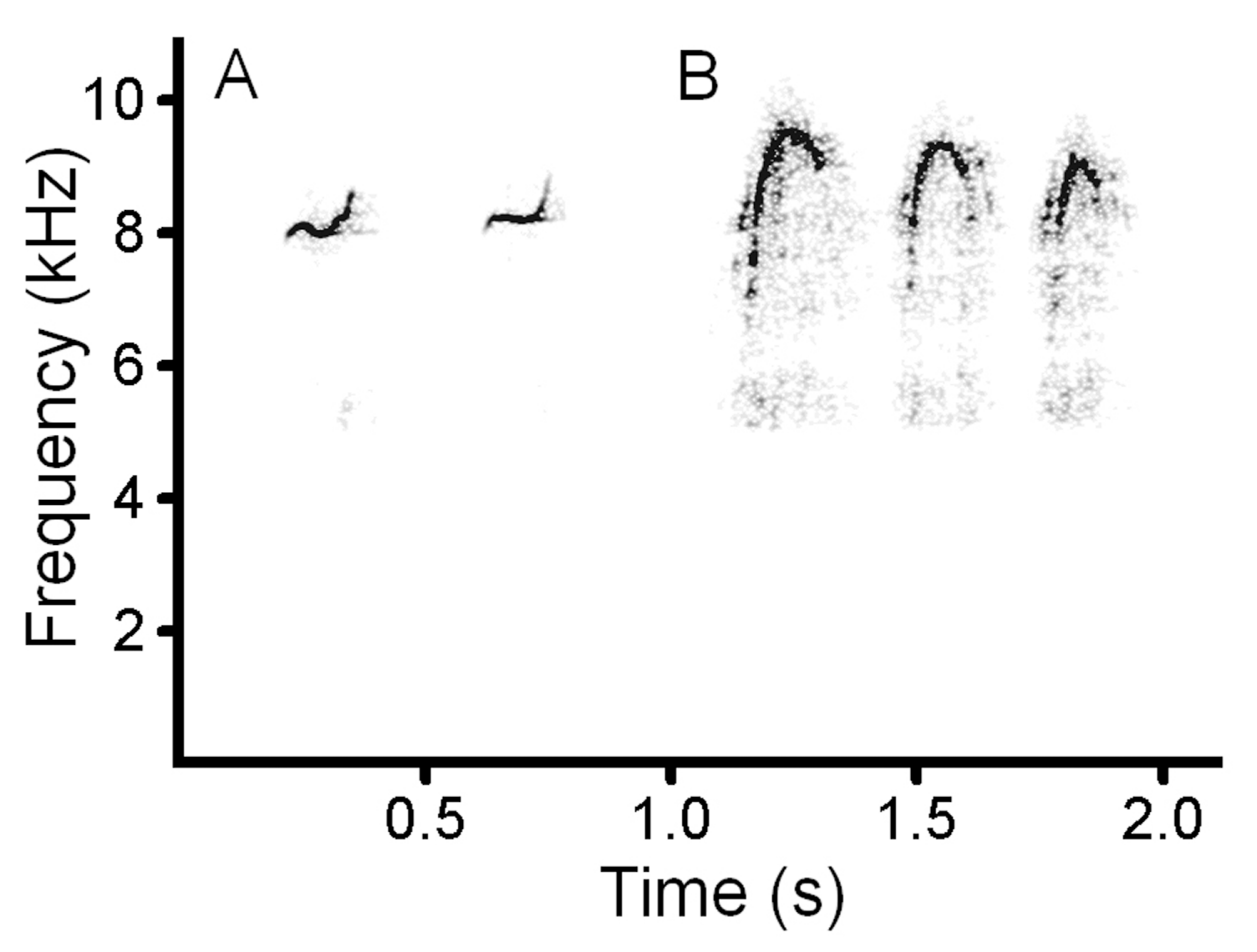
Spectrogram illustrating the acoustic similarity between the high-frequency squeaks of a dhole (A) and the whistles of a human (B). (A) A natural series of a captive male dhole. (B) A natural series of an adult male zoo visitor, imitating the dhole calls (S4 Audio). A 5 kHz high-pass filter was applied to remove background noise. Spectrogram settings as in Fig 1.

We identified narrowings in the nasal vt as potential sites for origin of g0. The g0 in dhole calls may arise as a result of vortex shedding during exhalation at adjustable narrowings in the flexible rostral parts of the nasal vt. Before reaching the nostrils, an exhalatory/phonatory airstream from the larynx has to pass the flexible region rostral to the osseous nose parts. In this region, the straight and the alar folds, which are supported by flexible nasal cartilages, might be suitable structures for g0 production (Fig 10). Approaching the two flexible folds by muscular action can be expected to create an obstruction in the rostral nasal air passage during exhalation through the nose that is sufficient to initiate vortex shedding. Depending on the degree of narrowing, the frequencies produced by this non-laryngeal mechanism may vary to a certain extent. In this case, a coupled and simultaneous adjusting of both nostrils would be necessary for producing a single g0. As evidenced by cineradiography, domestic dogs produce their high-frequency squeaks through the nose, with a lowered soft palate and with the laryngeal entrance protruding through the intra-pharyngeal ostium into the nasopharynx, i.e. with an ‘intra-narially positioned’ epiglottis [38]. Indirect evidence comes from the acoustic similarity between high-frequency squeaks of dholes and their imitation by human whistles (Fig 12). The production of whistles, although emitted through the mouth, involves vortex shedding, and the created frequency depends on the amount of narrowing of the lips [72]. Further support for this mechanism in the dhole is provided by the identification of vortices at vt narrow apertures as a source of the 22- and 50- kHz vocalizations in rats *Rattus norvegicus* [73,74].

Vorticity-induced vibrations of the vocal folds resulting from the interaction of vt vortices with vt resonances might exert a periodic lifting force on the vocal folds and provoke the appearance of a second frequency in the call spectrum. This mechanism of biphonation might be relevant for the dhole, as a narrow laryngeal vestibule enhances the source-tract interactions and amplifies glottal instabilities [34,39,40]. The sound wave, originating from these vortices, will propagate from its point of origin both forward and backward along the vt, i.e. towards the nostrils and towards the larynx. This can be expected to result in the appearance of a biphonic sound.

#### Vestibular folds

The production of g0 may result from high-frequency oscillations of the short vestibular folds that were about half the length of the vocal folds in the dhole, as in red fox and in domestic dogs [30]. Considering the small length of the vestibular folds, they should potentially be able to produce a second fundamental frequency g0 that is pronouncedly higher than the low fundamental frequency f0 produced with the two times longer vocal folds. Production of the high frequency oscillations would require a tensioning of the vestibular folds. However, in contrast to the domestic dog [23,30], the thyroarytenoid muscle of the dhole is not subdivided into a ventricularis and a vocalis muscle (S2 Table) and, thus, the lateral laryngeal ventricle (LLV) is not flanked rostrally and caudally by the two portions of the thyroarytenoid muscle. As a consequence, control of the vestibular fold by a separate ventricularis muscle cannot occur in the dhole. However, control of the vestibular fold’s tension may be achieved by alternative mechanisms. As the basic scaffolding of the larynx, consisting of the laryngeal cartilages and their inter-connecting ligaments, is a complex elastic system, any muscular contraction will cause a deviation from its intermediate resting position to some kind of “tensed” position and will return to the resting position after muscle contraction has ended. Considering the connection between the arytenoid cartilage and the cuneiform process, any positional change of the arytenoid cartilage will entail some change in position of the cuneiform process as well. Thus, the contraction of the lateral cricoarytenoid muscle alone or joint contractions of this muscle and the transverse arytenoid muscle may not only narrow the glottic cleft (S2 Table), but also exert some pulling on the cuneiform process and, thereby, may assist in regulating tension of the vestibular fold. As the dorsal end of the cuneiform process is connected with the lateral process of the epiglottis, any movements of the epiglottis will also influence the position of the cuneiform process. Ventral depression of the epiglottis by contraction of the hyoepiglottic muscle, as it may occur during a call, will pull the dorsal end of the cuneiform process ventrally and, thereby, pull its ventral end dorsally. This can be expected to apply tension to the vestibular fold and, thus, to influence its oscillation frequency [27].

However, oscillations involving the vestibular folds are usually located in the low-frequency range, both in excised canine larynges (90–93 Hz) [27] and during vocal exercises of humans (72 Hz) [75]. In our study, the g0 frequencies of the dholes were high, suggesting that oscillations of the vestibular folds are not involved in g0 production.

#### Knife effect

In contrast to the vocal folds, the edges of the vestibular folds are directed caudally and, therefore, are exposed to the exhalatory air stream from the glottis towards the mouth or nose in an opposite way (the edge is hit first and the rest of the fold later). This configuration of a sharp edge facing a counter-current air flow of relatively high speed may produce turbulent noise by the creation of vortices in the direction of the air flow, a principle called ‘edge tone’ [72].

In addition, the LLVs, the openings of which are exposed to the exhalatory air flow laterally adjacent to the vestibular folds (Fig 6), may separate a narrow band of frequencies from that turbulent noise and concentrate almost all sound energy on these frequencies [72]. This frequency band will correspond to the current resonance frequencies of the LLVs. These resonance frequencies of the LLVs can be expected to vary considerably with relevant parameters. The volume of the LLVs may vary in accordance with the amount of inflation, which will effect different resonance frequencies of the LLVs. Size changes of the LLVs may effect some kind of tuning of their resonance frequencies. Different degrees of inflation may be regulated by tensioning and relaxing of the vestibular folds. Contractions of the thyroarytenoid muscle, that covers the opening and ‘neck’ of the LLV laterally, will decisively influence the tension of the vestibular ligament and the size of the entrance to the LLVs. The angle of the vestibular fold relative to the exhalatory air flow might vary as a consequence of differing positions of the ventral end of the cuneiform process. The variation of these parameters will essentially depend on the contraction status of those muscles operating the position of the arytenoid cartilage and, thereby (indirectly), the position of the cuneiform process (lateral cricoarytenoid muscle, transverse arytenoid muscle) (S2 Table).

The LLV could be seen as a functional convergence to a technical variable-frequency oscillator [76]. Interestingly, an African domestic dog breed with size-reduced LLVs, the Basenji dog, is incapable of barking and the voice is described as some kind of strange yodeling or not unlike a young cockerel’s first attempt at crowing [77]. This implies the basic significance of the LLVs for the quality of the produced sound as well as the shift of the sound to higher frequencies when the volume of the LLVs decreases.

#### Vocal membranes

All the 4 study dholes were capable of producing g0 and biphonation but none of these specimens possessed vocal membranes on their vocal folds. Apparently, the production of high-frequency vocalizations and biphonic calls in dholes do not depend on vocal membranes.

Taken together, our data do not support a mechanism of g0 production based on vocal membranes. A decision in favour of the remaining potential sources of high frequency and biphonic vocalization in the dhole cannot be made on the basis of this study. Definite identification of the source(s) would require experimental tests on live animals.

## Supporting Information

S1 Audio Domestic dog calls. Low-frequency whine, high-frequency squeak and biphonic whine-squeak.
(WAV)

S2 Audio Dhole calls. Low-frequency yap, high-frequency squeak and biphonic yap–squeak.
(WAV)

S3 Audio Red fox call. Low-frequency whine.
(WAV)

S4 Audio A natural series of two high-frequency squeaks of a captive male dhole followed by a natural series of three whistles of an adult male zoo visitor, imitating the dhole calls. (WAV)

S1 Table Acoustic measurements of 291 dhole calls. 104 yaps, 89 biphonic yap-squeaks, and 98 squeaks.
(XLS)

S2 Table Intrinsic laryngeal muscles of the dhole. (DOC)

S3 Table Extrinsic laryngeal, hyoid and lingual muscles and muscles of the fauces of the dhole. (DOC)

## List of abbreviations for the anatomical structures

App. hyo.: hyoid apparatus
Arc. zyg.: zygomatic arc
Art. cricthyr.: cricothyroid articulation
Art. thyroh.: thyrohyoid articulation
Basih.: basihyoid
Burs. phar. lat.: lateral pharyngeal pouch
Cart. aryt.: arytenoid cartilage
Cart. cric.: cricoid cartilage
Cart. intaryt.: interarytenoid cartilage
Cart. nas. lat. dors.: dorsolateral nasal cartilage
Cart. sesam.: sesamoid cartilage
Cart. thyr.: thyroid cartilage
Cav. Oris: oral cavity
Cerath.: ceratohyoid
Cerebr.: brain
Choan.: choanae
Con. nas. dors.: dorsal nasal concha
Con. nas. ventr.: ventral nasal concha
Cont. ventr. lar.: contour of lateral laryngeal ventricle
Cornu rostr.: rostral horn of thyroid cartilage
Cost. I: first rib
Epigl.: epiglottis
Epih.: epihyoid
Gl. thyr.: thyroid gland
Lig. cricthyr.: cricothyroid ligament
Lig. voc.: vocal ligament
Ling.: tongue
Man. stern.: sternal manubrium
Meat. ac.: acoustic meatus
Meat. nas. med.: middle nasal duct
Meat. nas. ventr.: ventral nasal duct
M. cricaryt. dors.: dorsal cricoarytenoid muscle
M. cricaryt. lat.: lateral cricoarytenoid muscle
M. cricphar.: cricopharyngeal muscle
M. cricthyr.: cricothyroid muscle
M. geniogl.: genioglossus muscle
M. genioh.: geniohyoid muscle
M. hyoepigl.: hyoepiglottic muscle
M. hyogl.: hyoglossal muscle
M. hyophar.: hyopharyngeal muscle
M. myloh.: mylohyoid muscle
M. sternoh.: sternohyoid muscle
M. sternthyr.: sternothyroid muscle
M. thyroaryt.: thyroarytenoid muscle
M. thyroh.: thyrohyoid muscle
M. thyrphar.: thyropharyngeal muscle
Nasophar.: nasal part of pharynx
N. vagus: vagus nerve
Nostr.: nostril
O: M. cricthyr.: origin of cricothyroid muscle
Oesoph.: oesophagus
Orophar.: oral part of pharynx
Ost. intrphar.: intra-pharyngeal ostium
Pal. dur.: hard palate
Pal. mol.: soft palate
Phar.: pharynx
Plan. nas.: nasal plate
Plic. ala.: alar fold
Plic. aryepigl.: aryepiglottic fold
Plic. bas.: basal fold
Plic. rec.: straight fold
Plic. vest.: vestibular fold
Plic. voc.: vocal fold
Proc. corn.: corniculate process of arytenoid cartilage
Proc. cun.: cuneiform process of arytenoid cartilage
Proc. musc.: muscular process of arytenoid cartilage
Proc. voc.: vocal process of arytenoid cartilage
Rad. ling.: root of the tongue
Styloh.: stylohyoid
Synchondr. thyroh.: thyrohyoid synchondrosis
T1: first thoracic vertebra
Thyroh.: thyrohyoid
Trach.: trachea
Tymph.: tympanohyoid
Ventr. lar.: lateral laryngeal ventricle
Vest. oesoph.: vestibulum of oesophagus
1^st^ Cart. trach.: first tracheal cartilage
