# Supplementary material for: Potential Sources of High Frequency and Biphonic Vocalization in the Dhole (Cuon alpinus)
Source: PLoS One. 2016 Jan 5;11(1):e0146330. doi: 10.1371/journal.pone.0146330 (PMC4701476; doi:10.1371/journal.pone.0146330)
Supplement: S2 Table — (DOC) [file pone.0146330.s006.doc]

Table S1. Intrinsic laryngeal muscles of the dhole.

| Muscle | Origin | Termination | Specific features | Function |
| --- | --- | --- | --- | --- |
| M. cricothyroideus | lateroventral half of cricoid arch, its fibres take an oblique course from caudoventral to rostrodorsal | caudolateral part of thyroid lamina, ventrally adjacent to the caudal knob of the oblique line; its border of termination runs obliquely from caudodorsal, close to the caudal horn of the thyroid cartilage, to rostroventral, covering a triangular ventrocaudal area of the thyroid lamina | caudally, its termination is covered by the sternothyroid muscle and, rostrally, by the thyrohyoid muscle, which terminate on and originate from the caudal knob of the oblique line of the thyroid cartilage, respectively; thick, powerful muscle  Innervation: N. laryngeus cranialis | narrows the glottic cleft by pulling the arch of the cricoid cartilage towards the fixed thyroid cartilage; this elevates the rostral end of the cricoid lamina and, thereby shifting the arytenoid cartilage dorsally, elongates the vocal folds and sets them under tension |
| M. cricoarytenoideus dorsalis | cricoid lamina, from median crest to lateral and laterorostral edge, its fibres take an oblique course from caudomedial to rostrolateral | caudomedial and caudolateral aspects of the muscular process of the arytenoid cartilage | Innervation: N. laryngeus caudalis | widens the glottic cleft by pulling the muscular process of the arytenoid cartilage dorsomedially and, thereby, shifting its vocal process laterodorsally |
| M. cricoarytenoideus lateralis | laterorostrally from the edge of the cricoid arch, its fibres take an oblique, dorsorostrally and slightly laterally directed, course | ventral aspect of the muscular process of the arytenoid cartilage | Innervation: N. laryngeus caudalis | narrows the glottic cleft by pulling the muscular process of the arytenoid cartilage ventrally and, thereby, shifting the vocal process medially |
| M. arytenoideus trans-versus | rostromedial aspect of muscular process of arytenoid cartilage and from arcuate line, its fibres take a transverse course | dorsally, in the median plane, to contra interarytenoid and sesamoid cartilages | Innervation: N. laryngeus caudalis | narrows the glottic cleft in cooperation with the lateral cricoarytenoid muscle by approaching the arytenoid cartilages; may assist the dorsal cricoarytenoid muscle in widening of the glottic cleft by approaching the arytenoid cvartilages dorsally and, thereby, shifting the muscular processes laterally |
| M. thyroarytenoideus | uniformly, along the median line, from caudal half of dorsal aspect of thyroid cartilage plus the cricothyroid ligament; tapering markedly to about one fourth of its ventralmost rostrocaudal dia-meter, its fibres course dorsally | vocal process and arcuate line of arytenoid cartilage and dorsally on transverse arytenoid muscle | not divided into a rostral ventricularis muscle and a caudal vocalis muscle, it covers the knob-like ventral end of the cuneiform process and the caudal 'neck' of the laryngeal ventricle whereas the major rostral portion of the laryngeal ventricle remains uncovered; innervation: N. laryngeus caudalis | varies tension and, thereby, thickness of the vocal folds by differential contraction, regulates the fine-tuning of the vocal fold; assists decisively in complete closure of the glottic cleft by increasing its diameter during strong contraction |
