# Supplementary material for: Potential Sources of High Frequency and Biphonic Vocalization in the Dhole (Cuon alpinus)
Source: PLoS One. 2016 Jan 5;11(1):e0146330. doi: 10.1371/journal.pone.0146330 (PMC4701476; doi:10.1371/journal.pone.0146330)
Supplement: S3 Table — (DOC) [file pone.0146330.s007.doc]

**Table S2**. Extrinsic laryngeal, hyoid, lingual muscles and muscles of the fauces of the dhole.

| Muscle (portion) | Origin | Termination | Specific features | Function |
| --- | --- | --- | --- | --- |
| M. levator veli palatini | muscular process of tympanic part of petrosum of temporal bone, osseous part of auditory tube, medial to the tensor muscle of the soft palate | soft palate and palatine aponeurosis, medial to pteropharyngeal muscle | connects to its contralateral counter-part in the median plane | raises the soft palate |
| M. tensor veli palatini | muscular process of tympanic part of petrosum of temporal bone, osseous part of auditory tube, lateral to the levator muscle of the soft palate | soft palate and palatine aponeurosis, lateral to pteropharyngeal muscle | passes the pterygoid hamulus laterally | tenses the soft palate |
| M. palatinus | horizontal plate of palatine bone, ventral to choanae | caudal edge of soft palate, contacting the intrapharyngeal ostium | blends with palatopharyngeal muscle | shortens the soft palate |
| M. stylopharyngeus caudalis | medially from dorsal half of stylohyoid, its fibres slightly diverge towards the termination | lateral wall of pharynx, medially and 6-7 mm caudally to stylohyoid |  | dilates the pharynx |
| Mm. constrictores pharyngis rostrales (M. pterygopharyngeus) | caudal edge of pterygoid bone, between the levator muscle (medially) and the tensor muscle (laterally) of the soft palate, its fibres course caudodorsally | pharyngeal raphe, rostral to the termination of the caudal stylo-pharyngeal muscle |  | constricts rostral portion of pharynx |
| Mm. constrictores pharyngis rostrales (M. palatopharyngeus) | caudal edge of pterygoid bone, palatine aponeurosis, medial to pteropharyngeal muscle, its fibres intermingle with those of the palatine muscle | pharyngeal raphe, caudal to the termination of the pteropharyngeal muscle | its fibres course medial to the termination of the caudal stylo-pharyngeal muscle | sphincter of nasopharynx and intrapharyngeal ostium |
| M. hyopharyngeus | lateroventral half of thyrohyoid, its fibres diverge laterally and converge dorsally towards the termination | pharyngeal raphe |  | constricts middle portion of pharynx |
| Mm. constrictores pharyngis caudales (M. thyropharyngeus) | laterodorsal aspect of thyroid lamina, along oblique line, dorsally adjacent to termination of sternothyroid muscle and to origin of thyrohyoid muscle, its fibres converge dorsally | pharyngeal raphe |  | constricts caudal portion of pharynx together with the cricopharyngeal muscle |
| Mm. constrictores pharyngis caudales (M. cricopharyngeus) | laterodorsal half of cricoid arch, dorsally adjacent to cricothyroid muscle, caudally adjacent to termination of sternothyroid muscle, its fibres diverge dorsally | pharyngeal raphe |  | constricts caudal portion of pharynx together with thyropharyngeal muscle |
| M. ceratohyoideus | entire rostral edge of thyrohyoid | almost entire caudal edge of epihyoid and entire dorsal edge of ceratohyoid |  | reduces the angle between thyrohyoid and ceratohyoid, raises thyrohyoid and pulls larynx rostrally |
| M. hyoepiglotticus | basihyoid and ceratohyoid, its fibres take an oblique, caudodorsally directed course | rostromedially, on the ventral aspect of the epiglottic cartilage |  | restores resting position of epiglottis after deglutition, pulls epiglottis ventrally |
| M. occipitohyoideus | paracondylar process | dorsocaudal edge of stylohyoid |  | pulls stylohyoid and, thereby, root of the tongue and larynx, caudally |
| M. stylohyoideus | dorsocaudal end of stylohyoid, caudally adjacent to origin of styloglossus muscle, its parallel fibres course ventrally | basihyoid, dorsally adjacent to termination of sternohyoid muscle and to origin of hyoglossus muscle | weak band-like muscle, consisting of few fibres, surrounding the digastric muscle (not described) laterally at its tendinous intersection between rostral and caudal belly | raises basihyoid and larynx caudodorsally |
| M. mylohyoideus | medial surface of body of mandibula, along mylohyoid line, its caudal fibres course medially, its rostral fibres course mediorostrally | basihyoid and tendinous median raphe | its transverse fibres support the throat region hammock-like | raises floor of oral cavity and tongue |
| M. styloglossus | dorsorostral edge of stylohyoid, close to its connection with tympanic bulla, its fibres course ventrorostrally | entire lateral edge of tongue, up to lingual apex | long, slender muscle | retracts and shortens the tongue or turns the apex to one side |
| M. hyoglossus | basihyoid, rostral to termination of sternohyoid muscle, rostral edge of hyopharyngeal muscle, few fibres from caudal edge of stylohyoid | lateral portion of tongue, between genioglossus muscle (medially) and styloglossus muscle (laterally) | its fibres intrude into the tongue from caudally, antagonist of M. genioglossus | retracts and depresses the tongue |
| M. genioglossus | rostromedial surface of the body of the mandibula, caudally adjacent to the intermandibular connection, medially adjacent to origin of geniohyoid muscle | ventral portion of tongue, parallel to the median plane, medial to hyoglossus muscle, its fibres strongly diverge dorsally | paramedian position, touches lingual septum medially | protrudes the tongue, depresses longitudinal centre portion of tongue furrow-like |
| M. geniohyoideus | rostromedial surface of the body of the mandibula, caudally adjacent to the intermandibular connection | basihyoid, medial to hyoglossus muscle | covered by mylohyoid muscle | moves hyoid and tongue rostrally |
| M. sternohyoideus | dorsal surface of manubrium sterni and first costal cartilage, together with sternothyroid muscle | basihyoid | common origin with sternothyroid muscle, throughout its length united with its contralateral counterpart along the midline and initially also with sternothyroid muscle up to tendinous inscription | pulls basihyoid, root of the tongue, and larynx caudally |
| M. sternothyroideus | dorsal surface of manubrium sterni and first costal cartilage, together with sternohyoid muscle | caudal knob of the oblique line of thyroid cartilage, touching the origins of thyrohyoid and thyropharyngeal muscles | common origin with M. sternohyoideus up to tendinous inscription | pulls larynx caudally |
| M. thyrohyoideus | oblique line of thyroid cartilage, from rostral notch to caudal knob, touching termination of sternohyoid muscle and origin of thyropharyngeal muscle | caudal edge of thyrohyoid | short muscle, 20-25 mm in length | pulls hyoid and tongue caudally or, with fixed hyoid, the larynx rostrally |
